# Determining Inaccurate Coordinates in Electronic Data Collection for Surveillance and Immunization Supportive Supervision: A Case Study of Nigeria EPI Supportive Supervision Module

**DOI:** 10.3389/fdgth.2022.907004

**Published:** 2022-06-09

**Authors:** Isah Mohammed Bello, Godwin Ubong Akpan, Abdulsalam Yau Gital, Musa Iliyasu, Danlami Mohammed, Faysal Shehu Barau, Daniel Oyaole Rasheed, Erbeto Tesfaye Bedada, Sylvester Maleghemi

**Affiliations:** ^1^Inter-Country Support Team Office for East and Southern Africa, World Health Organization (WHO), Harare, Zimbabwe; ^2^Regional Office for Africa, World Health Organization (WHO), Brazzaville, Republic of Congo; ^3^Department of Computer Science, Abubakar Tafawa Balewa University, Bauchi, Nigeria; ^4^Department of Computer Science, Abubakar Tatari Ali Polytechnic, Bauchi, Nigeria; ^5^Nigeria Country Office, World Health Organization (WHO), Abuja, Nigeria; ^6^WHO Country Office, World, Health Organization (WHO), Juba, South Sudan

**Keywords:** Geographic Information System (GIS), immunization, vaccine-preventable disease, electronic data collection, coordinates accuracy

## Abstract

The mobile phone global positioning system (GPS) is used to reconnaissance a mobile phone user's location, e.g., at work, home, shops, etc. Such information can be used to feed data gathering expeditions, the actual position of the interviewer/surveyor using the mobile phone inert settings of location mode *via* GPS, WIFI, and Mobile networks. Mobile devices are becoming progressively erudite and now integrate diverse and robust sensors. The new generation of smartphones is multi-laden with sensors, including GPS sensors. The study describes and evaluates a data-gathering process used by the World Health Organization (WHO–Nigeria, EPI Program) that uses phone-based in-built GPS sensors to identify the position of users while they undergo supportive supervision. This form of spatial data is collected intrinsically using the Open Data Kit (ODK) GPS interface, which interlaces with the mobile phone GPS sensor to fetch the geo-coordinates during the process. It represents a step in building a methodology of matching places on the map with the geo-coordinates received from the mobile phones to investigate deviation patterns by devices and location mode. The empirical results can help us to understand the variation in geospatial data collation across devices and highlight critical criteria for choosing mobile phones for mobile surveys and data campaigns. This study reviewed the existing data gathered inadvertently from 10 brands of smartphones over 1 year of using the mobile data collection with over 80,000 field visits to predict the deviation pattern for spatial data acquisition *via* mobile phones by different brands.

## Introduction

The program managers and policymakers need accurate and timely data to improve the quality of their services. The demand for quality health data is high and used as input for vital decision-making processes that impact socioeconomic and environmental behavior monitoring. It serves as input to the health systems toward predicting health outcomes and reducing morbidity and mortality of diseases (1). The use of mobile phone-based systems and geospatial technologies for collecting such data is becoming one of the critical components of public health program implementation. Mobile data collection has been used for supportive supervision in the organization to merge the process of data collection and data entry. It replaced the paper-based manual method of data collection to conduct the supportive supervision and other surveys in the organization (2).

Geographic Information System (GIS) technology has improved our daily lives in various ways. International organizations can benefit tremendously from the increased incorporation of geospatial data and technologies into their operational systems (3). The inclusion of GIS technologies into their systems has dramatically increased the effectiveness of their processes and operations. Currently, most organizations are beginning to make effective use of only a minimal amount of geospatial data, most of which is collected on paper and prone to errors (4). GIS technologies and geospatial data can aid in the creation of quickly generated visual maps that can be used to guide local, regional, and global decision-making processes (5). Additionally, such data can serve as a guide to support decisions on where to move forward next in operations.

Historically, data collection was considered a colossal task, meant only for the GIS professionals and few technical persons regarded as the technical savvy crowd. This is because of the difficulty involved in learning and operating mapping grade global positioning system (GPS) equipment and systems. But recently, smartphones and tablets have become the dominant force that has simplified communication and GIS data collection globally. Due to increased usage for scientific investigation and other aspects of human endeavors (6), mobile phones have offered great and amazing functionalities that are more coherent and friendly. However, mobile data collection is not without problems of its own. Instances of having difficulty in usage and user-friendliness of the system (7), coordinates lying outside the focus region, low accuracy (8), satellite geometry, signal blockage, atmospheric conditions, etc., are common issues found with the system (9–11).

The Ebola Virus Disease outbreak in Nigeria in 2014–2015 changed the data landscape in the health sector. The country was forced to submit real-time data on each case and contacts to the Emergency Operations Center (EOC) for early detection and response. The Open Data Kit (ODK) and Form Hub technology were used in combination with the Dashboard technology and ArcGIS mapping for follow up of contacts, identification of cases, case investigation and management and also for strategic planning during the response (12). Lessons learnt were collated and brought into the routine immunization and surveillance systems *via* the integrated supportive supervision (ISS) system. The ISS is a checklist with a set of questions that are used at a health facility to monitor the surveillance and routine immunization processes, with additional functionalities for validation and verification, which includes coordinates locations, pictures, device ID as well as stamp date and time, that are collected during the supportive supervision visits (2). This study analyzed the data from the health facility based-supportive supervision module, which was conducted using different mobile phones by different WHO personnel in Nigeria. It investigates the deviation patterns according to devices and isolate records with inaccurate coordinates that are lying outside the focus region (Nigeria). The aim is to determine and investigate the reason behind the deviation and understand the factors influencing that based on the type and model of phone used and the cadre of personnel conducting the visits, this is helpful in identifying the best and most appropriate mobile device to be used for data collection during supportive supervision thus improving the performance.

## Methodology

### Study Design, Population, and Context

We conducted a secondary analysis of data generated by the ISS module from all the six geopolitical zones of Nigeria from January to December 2016. The study covers the Expanded Programme on Immunization (EPI) health facility-based supportive supervision module for WHO Nigeria country office.

Nigeria is located in the West Africa on the Gulf of Guinea between Benin and Cameroun, with an estimated population in 2018 of 198 million persons as projected from the 2006 Census. It comprises 36 states and the Federal Capital Territory, with the states organized into six (6) geopolitical zones. Immunization coverage varies across Nigeria with improvements needed in every state, as no state meets the global coverage of 90% coverage for the three doses of pentavalent vaccine. The immunization coverage is least in the Northern zones and mitigated by having more WHO staff to support the ministry of health (MOH) EPI activities, with high coverage reported from the South–West zone (13). The WHO personnel implemented the use of android-based mobile data collection using the ODK for supportive supervision with 81,505 records available on the server, corresponding to 81,505 ISS visits to health facilities. All data from the 36 states plus federal capital territory (FCT) (36 + FCT) in the country were selected and included in the study.

### System for Collection of Surveillance and Routine Immunization Supportive Supervision Data in Nigeria

The ISS checklist was translated from the paper format into ODK format and uploaded on mobile phones for users to conduct supportive supervision. The users visit a health facility to conduct supportive supervision through a combination of different strategies (interview, record review, observation, and probing/validation, etc.), and collect information regarding routine immunization and vaccine-preventable disease surveillance processes. The user then saves the information in his/her mobile phone and then send the data to a central server, which has the additional features of analytics with a dashboard created to provide real-time analysis of the data stored to guide the program.

### Data Analysis

**Figure 1:**
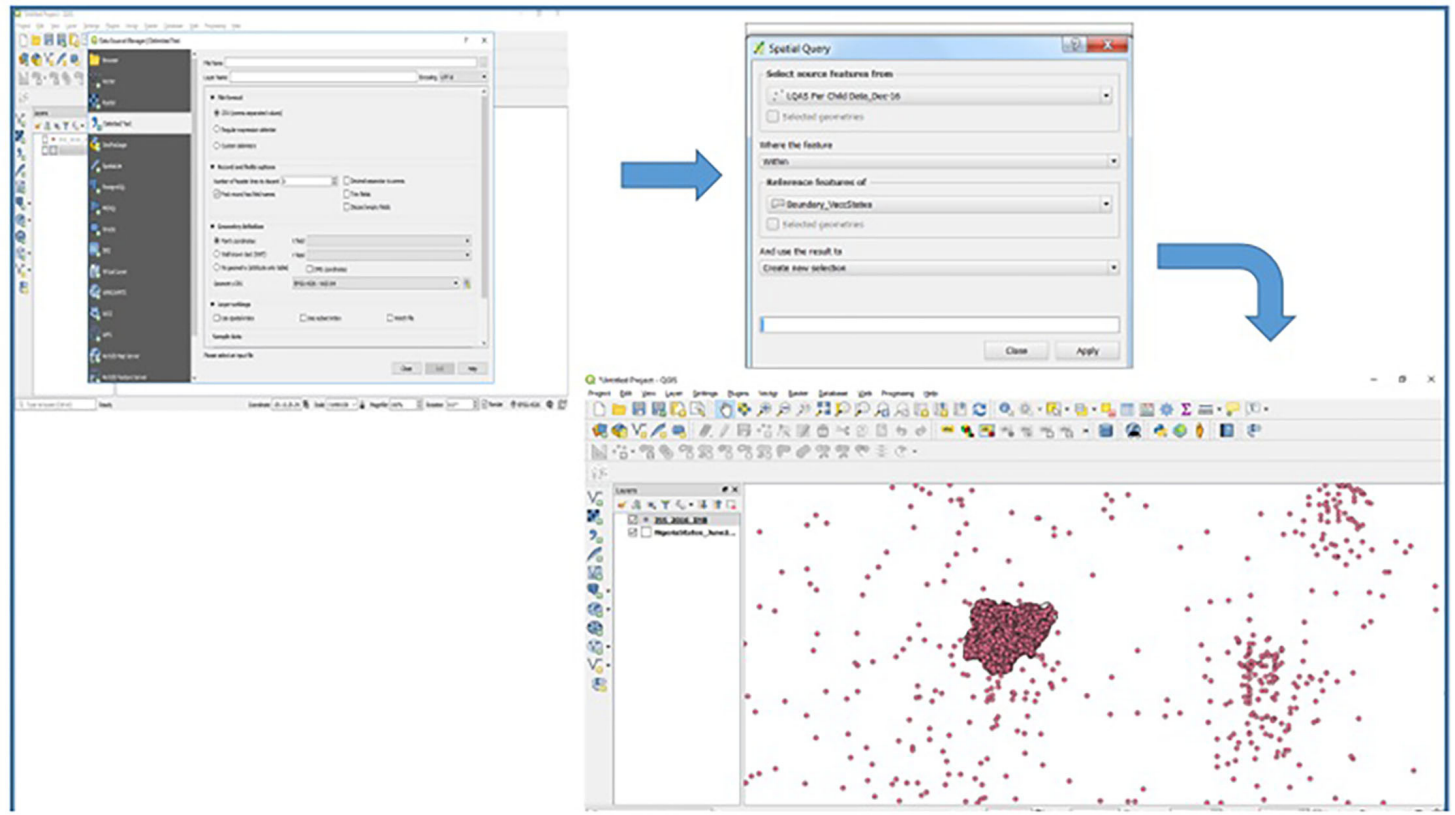
Spatial analysis using Quantum Geographic Information System (QGIS) to extract integrated supportive supervision (ISS) records with inaccurate (skewed) coordinates.

The ISS visits conducted within the period January–December 2016 were analyzed using spatial analysis on the Quantum Geographic Information System (QGIS) to extract records, which are lying outside their focus areas (inaccurate coordinates). The data were downloaded in a comma-separated values (CSV) format, imported into the QGIS applications, and displayed as X and Y coordinates. The information was displayed against the state boundaries using the Nigeria's country shapefile. The records lying outside the boundaries were extracted using the spatial query as shown in Figure 1. The extracted data outside the boundaries were then saved as shapefiles, which were then extracted as an excel file from the MDB file generated from the shapefile. The extracted data have a field for device ID that was used to determine the type, model of the device used to conduct the supportive supervision, which was further disaggregated by geopolitical zone/states and cadre of the personnel. The device ID was then used to find the type and model of the phone using the IMEI site (https://www.imei.info).

## Result

During the period under review, a total of 81,505 ISS visits were conducted across the 36 states (36) plus the FCT, as presented below in Table 1.

**Table 1:** Number of records and those with inaccurate coordinates by political zone and cadre of personnel.

**Zones**	**No of states**	**No. and percentage of records on the server [N, %]**	**No. and percentage of records with inaccurate coordinates on the server (N, %)**	**No. and percentage of records by State Coordinators (SCs) on the server**	**No. and percentage of records by Cluster Coordinators (CCs) on the server**	**No. and percentage of records by LGA Facilitators (LGAF) on the server**
North-Central	7	12,986 (16)	123 (0.9)	81 (0.6)	4,433 (34.1)	8,472 (65.2)
North East	6	21,691 (27)	235 (1.1)	64 (0.3)	6,660 (30.7)	14,967 (69.0)
North-West	6	36,477 (45)	652 (1.8)	56 (0.2)	7,750 (21.2)	28,671 (78.6)
South-East	5	1,860 (2)	12 (0.6)	98 (5.3)	416 (22.4)	1,346 (72.4)
South-South	6	4,464 (5)	52 (1.2)	114 (2.6)	1,119 (25.1)	3,231 (72.4)
South-West	7	4,027 (5)	45 (1.1)	103 (2.6)	826 (20.5)	3,098 (76.9)
Total	37	81,505 (100)	1,119 (1.4)	516 (0.6)	21,204 (26.0)	59,785 (73.4)

A total of 1,119 records were found to have inaccurate coordinates, representing 1.4% of the total records. The North West Zone had the highest number of records with 36,477, with 652 having inaccurate coordinates (1.8%), while the South East Zone had the lowest number of ISS visits with 1,860, and 12 records with inaccurate coordinates (0.6%). Most of the visits were conducted by the Local Government Area Facilitators (LGAF), having over 73% of the total visits, with all the zones having more than 65% for the LGAF category. The Cluster Coordinators (CC) category contributed 26% of the visits, and all the zones are within the range of 20–34% of the total visits, while the state coordinators (SC) had only 0.6% of the total visits, ranging from 0.2% in North–West to 5.3% in South–East.

Out of the total visits conducted, eight different brands of mobile devices were used to conduct the visits, with 40 models represented. Table 2 depicts the different brands of mobile phones used to conduct the supportive supervision visits during the period under review. Samsung had the highest number of models and records with 14 different models and 59,534 records representing 73% of the total visits and had only 1.4% records with inaccurate coordinates. HTC has two (2) models, with the lowest number of records of 80 (0.1%) and had no records with inaccurate coordinates. The HUAWEI brand is second with the highest records, 19,977 (24.5%), with only three (3) models and a 1.1% proportion of records with inaccurate coordinates. However, the iTEL brand had 130 records, representing only 0.2%, but with a 63.1% proportion of records with inaccurate coordinates across three (3) different models.

**Table 2:** Number of records and those with inaccurate coordinates by mobile phone brand.

**Brand**	**No of models**	**No of records on the server (n)**	**% records on the server (%)**	**No. and percentage of records with inaccurate coordinates (n)**	**% of records with inaccurate coordinates (%)**
HUAWEI	3	19,977	24.5	220	1.1%
SAMSUNG	14	59,534	73.0	814	1.4%
Infinix	4	192	0.2	1	0.5%
TECNO	8	744	0.9	1	0.1%
HTC	2	80	0.1	0	0.0%
iTel	3	130	0.2	82	63.1%
GIONEE	5	748	0.9	1	0.1%
MOTOROLA	1	100	0.1	0	0.0%
Total	40	81,505	100.0	1,119	1.4%

From the total number of records with inaccurate coordinates, Table 3 shows that most of the records with inaccurate coordinates were from the LGAFs, having over 80.6% of the total inaccurate coordinates. The SC category had 0.6%, while the CC category had 18.8%. North–Central, North–East, North–West, and South–East have the majority of the inaccurate coordinates from LGAF with 85.4, 82.6, 80.4, and 100%, respectively, while South–South and South–West have 67.3 and 71.1%, respectively.

**Table 3:** Number of records and those with inaccurate coordinates by political zone and cadres of officers reporting.

**Zones**	**Total No of records with inaccurate coordinates (N)**	**No. and percentage of records with inaccurate coordinates by State Coordinators (SCs) [N, %]**	**No and percetnage of records with inaccurate coordinates by Cluster Coordinators (CCs) [N, %]**	**No. and percentage of records with inaccurate coordinates by LGA Facilitators (LGAFs) [N, %]**
North-Central	123	1 (0.8)	17 (13.8)	105 (85.4)
North East	235	3 (1.3)	38 (16.2)	194 (82.6)
North-West	652	2 (0.3)	126 (19.3)	524 (80.4)
South-East	12	0 (0.0)	0 (0.0)	12 (100)
South-South	52	0 (0.0)	17 (32.7)	35 (67.3)
South-West	45	1 (2.2)	12 (26.7)	32 (71.1)
Total	1,119	7 (0.6)	210 (18.8)	902 (80.6)

## Discussion

To strengthen the accountability within the RI and surveillance system, WHO staff across Nigeria piloted the use of ODK for ISS visits, to improve transparency and increase donor trust through documented and verifiable evidence with geo coordinates location, and using a standard checklist for uniformity and ease of data analysis. The findings from the pilot were used to improve the ISS checklist forms on the ODK and were scaled up for the utilization by the MOH and immunization at all levels of the country. The WHO Nigeria has personnel presence in all states, with LGA facilitators working at the local government level and reporting to CC who reports to the state coordinators (SC). The Who personnel provide support to primary healthcare, and regularly conduct supervisory visits to health facilities, using ODK application loaded on mobile devices.

The introduction of the ODK for ISS visits started from the Northern part of Nigeria, with few visits agreed to be conducted by all personnel, but as the system improves, an accountability framework was designed with a fixed number of visits by each cadre of personnel by month; LGAFs (9), CCs (6), and SCs (3). These visits were used as part of the performance evaluation of staff on a quarterly basis with feedback given to each person on their performance and use of the checklist (2).

The Northern part of the country, specifically the North–West zone, had the highest records, unsurprisingly as the bulk of WHO personnel are in the Northern part of the country, which is endemic for the wild poliovirus, thereby supporting the polio eradication initiative (14, 15). The Southern part especially the South–East has the limited personnel, thereby contributing a minimal percentage to the total records on the server.

From the total number of visits recorded (81,505), a total of 1,119 (1.6%) represent the records with inaccurate coordinates, with the majority of the inaccurate coordinates coming from the North–West. However, further analysis showed that the overall percentage of records with inaccurate coordinates in all the zones are almost the same, with only the South–East and North–Central zones doing better than other zones (Table 1).

At the beginning of ODK's introduction for ISS visits, no phone brands were recommended by the organization, with personnel allowed to purchase and use any brand of their choice (2, 16). Eight (8) brands and 40 models were documented in this study, with Samsung being the most common, this may relate to the huge available number of Samsung models available in the country, a statistics of smartphones in the country between 2016 and 2019 showed that the Samsung had the highest share of the smartphone market in Nigeria with 23% (17). The iTel had the highest number of inaccurate coordinates with HTC, and Motorola having no inaccurate coordinates. This may be due to the quality of the phones, the skills and training provided for the users on the use of ODK, and how to collect geo-coordinates. Most of the iTel phone models used by the personnel documented in the study were low-end, and this may be the reason behind the high number of inaccurate coordinates.

The majority of the records submitted were from the LGAFs, as they remain the bulk of the WHO personnel in Nigeria with most LGAs having at least 1 LGA facilitator in the north. The LGAFs also recorded the highest number of inaccurate coordinates, as this group of personnel is the lowest cadre with limited or no adequate training on the use of ODK at the time of the study. The LGFS also receives low remuneration as compared to CCS and SCs, hence the possibility of buying cheaper and low-end mobile phones that are not able to capture coordinates easily. The SCs who are the technical leads, reported little or no inaccurate geo-coordinates as they were trained many times before the introduction and use of the ODK, and have more disposable funds to purchase high-end phone models. This is in line with the assertion that lack of training is one of the challenges of using mobile in data collection (18–20).

The South–East had the best records at all levels with mistakes only coming from the LGAFs, while the South–South and South–West CCs have a greater number of records with inaccurate coordinates as compared to the other zones (Table 1), which may be due to poor training or staff not being aware of the importance of the geocoordinates. It is also pertinent to note that the use of mobile phones for data collection had become commonplace with many studies documenting the challenges of training, lack of patience in collecting coordinates location, and the capability of the mobile phones or tablets used in data collection (19, 21–23), which is similar to the result found in this study, sighting training and the use of the right mobile device while implementing mobile data collection.

One of the key components of mobile data collection is the need for setting a minimum requirement for any phone to be used in conducting supportive supervision visits, and if possible the organization can procure phones that had shown to have the least proportion of inaccurate coordinates to be used by the personnel and avoid the bring your own device (BYOD) policy. We are also recommending training to all staff carrying out this activity on the basic use of phones, taking geo-coordinates, and scenarios that affects coordinates capture in the field.

Our study examined the records with inaccurate coordinates from the supportive supervision checklist using mobile data collection based on the model and type of phones used, the cadre of the personnel conducting the visits. But there may be other factors influencing the recording of inaccurate coordinates for mobile data collection, which may require further studies to explore. Another limitation of this study is the lack of information on the training level of the different cadres of personnel that are utilizing the checklist; there are also issues related to some coordinates lying outside the actual states or local government focus region, as our study is only limited to records that are lying within the country, and there might be possibilities of inaccurate coordinates lying outside states or Local government area boundaries.

## Conclusion

The use of mobile phone data collection in the implementation of ISS for the surveillance and immunization in Nigeria had ensured the availability of real-time data, which supports decision-making process to guide the program, it enhances the transparency and accountability of the processes, thereby making it an interesting approach, which has been extended to other similar programs across the organization and EPI program in general. However, lack of training and selection of the right mobile phone are some of the factors that needed to be improved toward reducing inaccurate coordinates location. We recommend that the organization procures and provides the needed mobile devices to their staff to conduct this activity and avoids allowing personnel to use their own devices. This process can be adopted for all relevant programs that require data collection in similar settings, as the benefits of mobile data collection using ODK, in terms of precise coordinate location and ease of use, makes it a favorable tool that should be further explored.

### What Is New?

There are many studies on the use of mobile data collection in improving health data; however, this study explored the details of the factors affecting the accurate collection of coordinates locations, and issues related to the type of mobile phone device to be used in data collection, which is one of the main advantages that the mobile data collection offers over the traditional paper-based system.

## Data Availability Statement

Publicly available datasets were analyzed in this study. Data can be provided on request.

## Author Contributions

IB, GA, AG, MI, FB, DM, DR, EB, and SM contributed to the conceptualization of the study and led the drafting of the manuscript. All authors reviewed and approved the final manuscript and contributed to the review of the literature.

## Conflict of Interest

The authors declare that the research was conducted in the absence of any commercial or financial relationships that could be construed as a potential conflict of interest.

## Publisher's Note

All claims expressed in this article are solely those of the authors and do not necessarily represent those of their affiliated organizations, or those of the publisher, the editors and the reviewers. Any product that may be evaluated in this article, or claim that may be made by its manufacturer, is not guaranteed or endorsed by the publisher.
